# Supplementary material for: Intranasal Administration of Forskolin and Noopept Reverses Parkinsonian Pathology in PINK1 Knockout Rats
Source: Int J Mol Sci. 2022 Dec 30;24(1):690. doi: 10.3390/ijms24010690 (PMC9820624; doi:10.3390/ijms24010690)
Supplement: Supplementary file 1 [file ijms-24-00690-s001.zip › ijms-2087628-supplementary.pdf]

SUPPLEMENTAL MATERIALS

Intranasal Administration of Forskolin and Noopept reverses Parkinsonian pathology in PINK1 knockout rats

Ruben K. Dagda [1,2,*,†], Raul Y. Dagda [1,2,†], Emmanuel Vazquez-Mayorga [1], Bridget Martinez [1] and Aine Gallahue [1,2]

1 Department of Pharmacology, Reno School of Medicine, University of Nevada, Reno, NV 89557, USA
2 CNS Curative Technologies LLC, 450 Sinclair Street, Reno, NV 89501, USA
* Correspondence: rdagda@med.unr.edu; Tel.:+1-775-784-4121
† These authors contributed equally to this work.

SUPPLEMENTAL FIGURES

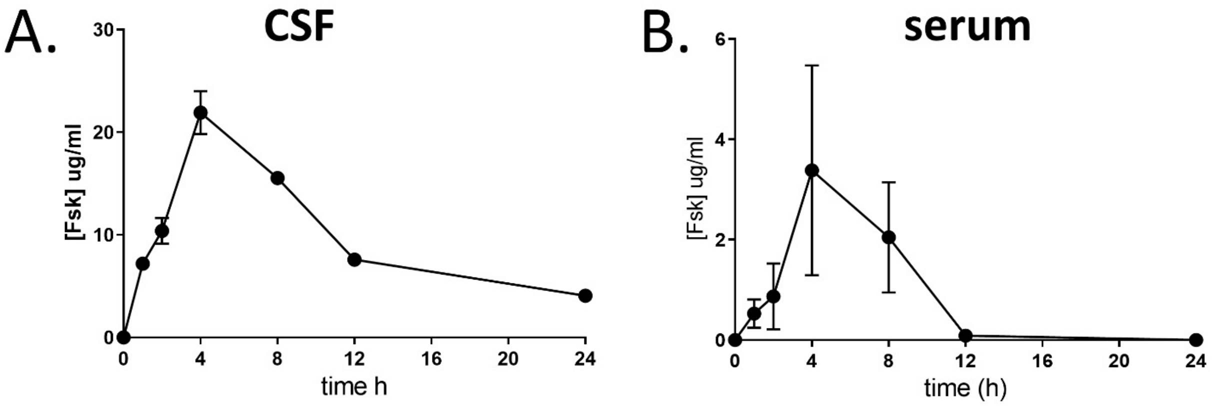

Figure S1. Pharmacokinetic analysis of Forskolin in rabbits intranasally treated with CNS/CT-001 **(a)** Compiled data on HPLC-mediated quantification of Forskolin the CSF of rabbits collected at the indicated time points following intranasal treatment with CNS/CT-001. **(b)** Compiled analysis of HPLC-mediated quantification of Forskolin in the serum of the same cohort of rats as in **(a)** at the indicated time points following intranasal administration of CNS/CT-001 with Forskolin and Noopept delivered at 166µM and 20nM respectively.

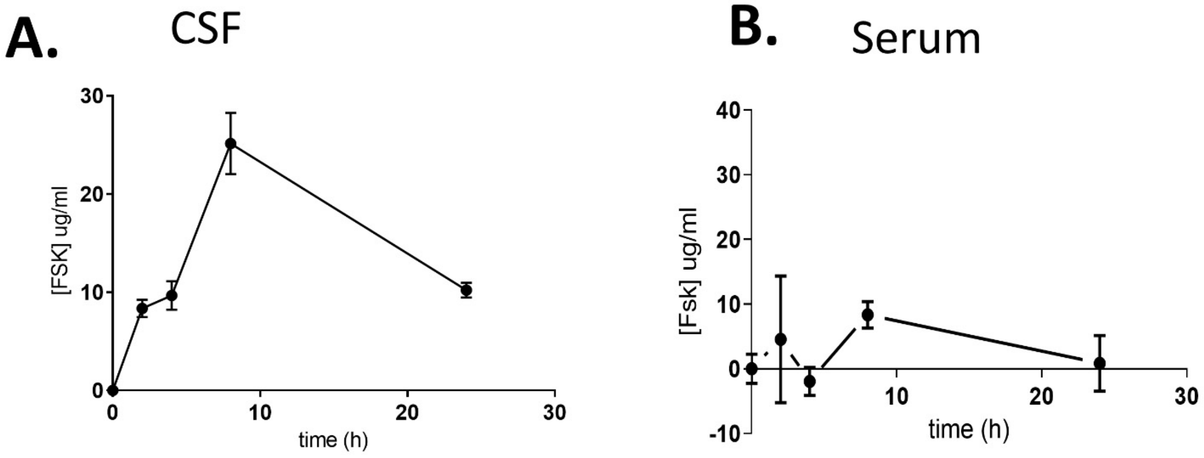

Figure S2. Pharmacokinetic analysis of Forskolin in rabbits intranasally treated with CNS/CT-001 **(a)** Compiled data on HPLC-mediated quantification of Forskolin in the CSF of rabbits collected at the indicated time points following intranasal treatment with CNS/CT-001 for one dose. **(b)** Compiled data on HPLC-mediated quantification of Forskolin in the serum of the same cohort of rabbits as in **(a)** at the indicated time points following intranasal administration of CNS/CT-001 with Forskolin and Noopept delivered at 166µM and 20nM respectively.

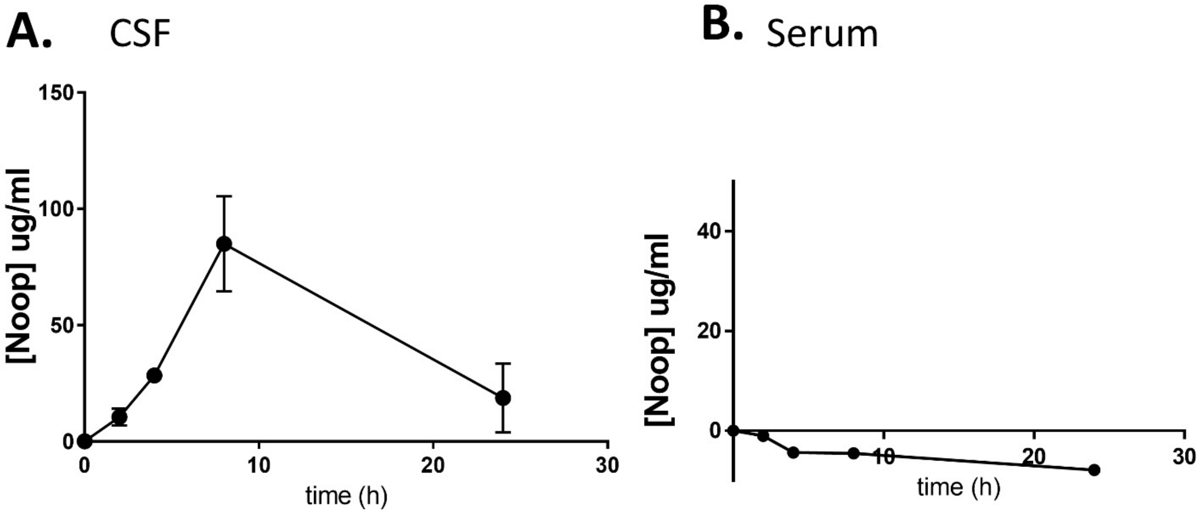

Figure S3. Pharmacokinetic analysis of Noopept in rabbits intranasally treated with CNS/CT-001 **(a)** Compiled data on HPLC-mediated quantification of Noopept in the CSF of rabbits collected at the indicated time points following intranasal treatment with CNS/CT-001. **(b)** Compiled data on HPLC-mediated quantification of Noopept in the serum of the same cohort of rabbits as in (a) at the indicated time points following intranasal administration of CNS/CT-001 with Forskolin and Noopept delivered at 166µM and 20nM respectively.

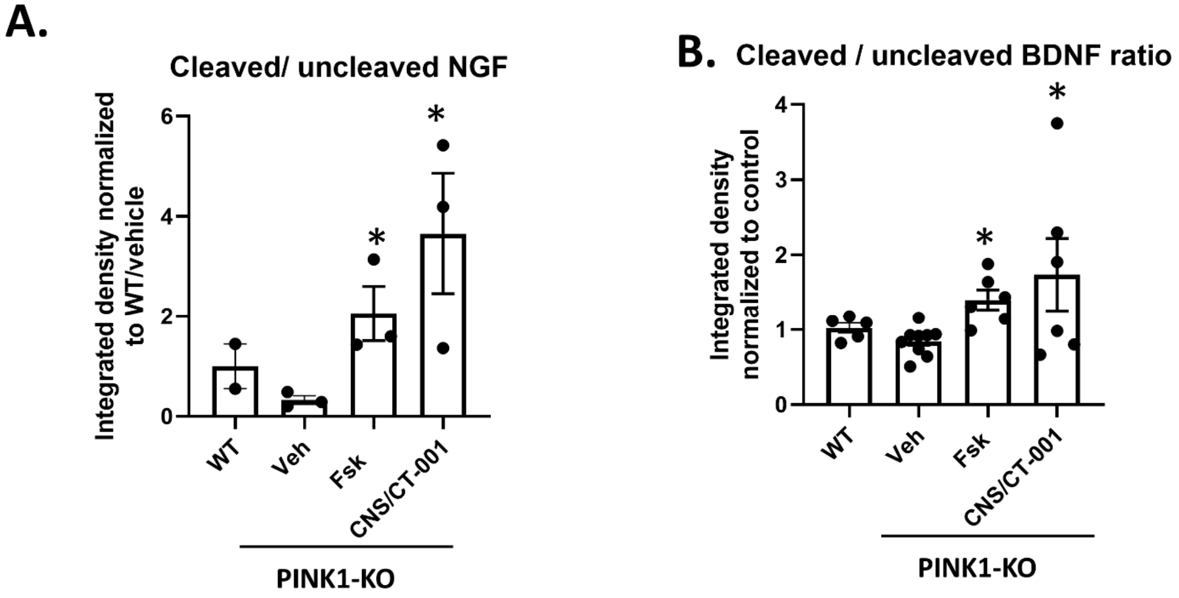

Figure S4. Intranasal administration of CNS/CT-001 elevates the levels of mature forms of neurotrophic factors in the cortex **(a)** Graph showing densitometric analysis of the mean quantification of the ratio of the integrated densities of cleaved to uncleaved immunoreactive bands for NGF as assessed by carrying out Western blot analysis of brain lysates from WT rats that were treated with vehicle solution, or 10-month-old PINK1-KO rat treated with the vehicle solution, with 20µM of Forskolin or treated with CNS/CT-001. **(b)** Graph showing densitometric analysis of the mean quantification of the ratio of the integrated densities of cleaved to uncleaved immunoreactive bands for BDNF as assessed by performing Western blot of brain lysates derived from WT rats treated with vehicle solution, or from PINK1-KO rat treated with the vehicle solution, intranasally treated with Forskolin (10µM) or treated with CNS/CT-001. For both (a) and (b), Mean ± SEM. *$p \leq 0.05$, One-Way ANOVA followed by the Kruskal-Wallis test, 3-8 animals per group). *:$p < 0.05$ vs. PINK1-KO/Veh (not significant by Bonferroni correction).
